# Attention Deficit/Hyperactivity Disorder Symptoms and Cognitive Abilities in the Late-Life Cohort of the PATH through Life Study

**DOI:** 10.1371/journal.pone.0086552

**Published:** 2014-01-28

**Authors:** Debjani Das, Nicolas Cherbuin, Simon Easteal, Kaarin J. Anstey

**Affiliations:** 1 John Curtin School of Medical Research, The Australian National University, Canberra, Australian Capital Territory, Australia; 2 Centre for Research on Ageing, Health and Wellbeing, The Australian National University, Canberra, Australian Capital Territory, Australia; University Children’s Hospital Tuebingen, Germany

## Abstract

Attention Deficit/Hyperactivity Disorder (ADHD) is a common neuropsychiatric disorder that has not been well studied in older adults. In this study we examined relationships between ADHD symptoms and cognitive ability and compared them between middle-age (MA; 48–52 years) and older-age (OA; 68–74 years) adults sampled from the same population. ADHD, mood disorder symptoms and cognitive abilities were assessed in a large population-based sample (n = 3443; 50% male). We measured current ADHD symptoms using the adult ADHD Self-Report Scale (ASRS), which we found to have the same underlying structure in both cohorts. Older adults reported significantly lower levels of ADHD symptoms and 2.2% of the OA cohort scored equal or above the ASRS cut-off score of 14 (which has been previously associated with ADHD diagnosis) compared with 6.2% of MA adults. Symptom levels were not significantly different between males and females. Using multi-group structural equation modelling we compared ADHD symptom–cognitive performance relationships between the two age groups. Generally higher ADHD symptoms were associated with poorer cognitive performance in the MA cohort. However, higher levels of inattention symptoms were associated with better verbal ability in both cohorts. Surprisingly, greater hyperactivity was associated with better task-switching abilities in older adults. In the OA cohort ADHD symptom–cognition relationships are indirect, mediated largely through the strong association between depression symptoms and cognition. Our results suggest that ADHD symptoms decrease with age and that their relationships with co-occurring mood disorders and cognitive performance also change. Although symptoms of depression are lower in older adults, they are much stronger predictors of cognitive performance and likely mediate the effect of ADHD symptoms on cognition in this age group. These results highlight the need for age-appropriate diagnosis and treatment of comorbid ADHD and mood disorders.

## Introduction

Attention Deficit/Hyperactivity Disorder (ADHD) is a neuropsychiatric disorder that develops in childhood and can persist throughout life. It is characterised by age-inappropriate levels of inattention and/or excessive motor activity and impulsivity [1]. In adults inattention is the predominant symptom, which manifests as disorganization, forgetfulness, unreliability, and poor performance in planning, task completion, task shifting and time management. ADHD symptoms affect functioning in multiple life domains often leading to employment and financial difficulties, and interpersonal problems [2], [3]. ADHD is associated with cognitive impairment in both children and adults [4]–[10]. Furthermore, individuals with ADHD are more likely than unaffected individuals to experience comorbid psychiatric disorders [2], [11]. The majority of adult with ADHD have at least one lifetime psychiatric comorbidity including anxiety (47%), mood (38%), impulse control (20%) and substance use disorders (15%) [12]. The high level of comorbidity has raised questions about whether the functional impairments in ADHD patients are primarily due to ADHD or the comorbid conditions. Addressing this issue a recent study reported that neuropsychological deficits in ADHD adults go beyond comorbidity [13]. The financial burden of ADHD on families and society is substantial due to direct medical expenses and the indirect costs of workplace productivity loss and accidents [14]–[18]. Furthermore, the negative impact of ADHD symptoms/impairments on professional, economic, social, and emotional well-being accumulates through life affecting quality of life at late-age [19].

### ADHD Prevalence in Late-life

ADHD is a common disorder affecting ∼5% of children and 1–7% of adults [20]–[25]. The prevalence of the disorder is thought to diminish with age [3]. However, very little is known about ADHD in late life since most studies on adult ADHD have focussed on young adults. Kooij et al (2005) [26] estimated the prevalence of ADHD among older adults (18–75 years) in the Netherlands to be 1–2.5%. The study by Michielsen et al. (2012) [27] reported prevalence rates of 2.8% and 4.2% for syndromatic and symptomatic ADHD, respectively, in adults aged 60–94 years. It is important to note that diagnostic criteria currently used for ADHD were developed for children and adolescents. It is uncertain how well these diagnostic criteria and thresholds apply to adults since the symptom patterns change with age [3]. Following recent recommendations [28], symptom threshold have been lowered for adults in the Diagnostic and Statistical Manual of Mental Disorders (5^th^ ed; DSM-5; [29]). Furthermore, DSM-based diagnosis of adult ADHD requires symptoms to be present in childhood – before 7 years in DSM-IV and before 12 years in DSM-5. Hence, diagnosis of ADHD in adults depends on accurate recall of childhood symptoms, which can be challenging for older adults. Thus, while it is accepted that ADHD persists throughout life, the condition is not well understood in old age and its prevalence is uncertain.

### ADHD Dimensionality

Although ADHD remains a categorically distinct clinical condition, there is increasing recognition that clinical ADHD lies at the extreme ends of the distribution of continuous ADHD symptom dimensions. Furthermore, symptoms of ADHD are common and can lead to functional impairment in adults who do not meet all the criteria for clinical diagnosis [2], [30]. A dimensional view is supported by evidences from several taxonomic, genetic and neuroimaging studies [31]–[34]. The majority of these studies are of childhood ADHD, but a recent taxonomic study in a sample of young adults also indicates a dimensional latent structure of ADHD in adults [35]. It is also well-established that ADHD is characterised by two related but distinct dimensions – inattention and hyperactivity [36], which have distinctive brain and cognitive characteristics [37]–[39]. The specific behavioural and cognitive characteristics of these dimensions are poorly understood. Their relative importance changes with age, with inattention becoming more dominant in adults, which may have an important impact on how the cognitive effects of ADHD change with age.

### Cognitive and Functional Consequence of Adult ADHD

Cognitive deficits have been consistently identified in both children and adults with ADHD [4]–[10]. Compared to unaffected individuals ADHD adults are cognitively different across multiple functional domains, with notable differences in attention, behavioural inhibition and non-executive functional aspects of memory, processing speed and motor speed [40]–[42]. Meta-analyses have also reported slightly lower IQ in adults with ADHD [40], [41], [43]. Neuropsychological research in ADHD has focussed primarily on aspects of attention and executive functions with multiple studies demonstrating deficits in vigilance, selective attention, distractibility, divided attention and flexibility, working memory, set shifting and planning [44]–[49]. Few such studies have been conducted in middle-aged or older adults. From a cross-sectional study of 116 non-medicated ADHD patients 19–55 years of age, Biederman et al. (2011) concluded that the negative impact of ADHD on cognition remains constant across the lifespan [50]. In another study, cognitive test profiles in older adults (62–91 years of age) were found to be associated with reports of childhood ADHD [51]. Using dimensional measures of ADHD symptoms we have previously reported that inattention and hyperactivity symptoms are associated with cognitive performance measures in middle-aged adults (47–54 years) [52]. Symptoms of inattention in particular were associated with poorer performance in this age group. Furthermore, some but not all of the effects of ADHD symptoms on cognition were mediated through co-occurring anxiety and depression symptoms. Thus, a very limited number of studies indicate that ADHD has a negative impact on cognitive functioning in old age, but the extent and nature of the impact is not well understood.

### The Present Study

In this study we extend our analyses of ADHD symptoms to a cohort of older adults (68–72 years) in the Personality and Total Health (PATH) Through Life Study, a population-based longitudinal study of mental health and ageing [53]. We focus on the association of ADHD symptoms with cognitive performance measures. To assess ADHD symptoms we used the adult ADHD Self-Report Scale (ASRS) [54], which is a checklist of inattention and hyperactivity symptoms based on the diagnostic criteria outlined in the DSM-IV-Text Revision. The ASRS demonstrated good sensitivity and specificity in clinical validation studies and is recommended for use in epidemiological studies [55]. However, the ASRS has not been validated in older adults. Hence, the first aim of the present study was to test whether the ASRS has the same underlying measurement properties in our middle-age (MA) and older-age (OA) cohorts. The second aim of the study was to examine whether relationships between ASRS latent factors (inattention and hyperactivity) and cognitive performance differ between the age groups. As a part of the second aim we also examined whether co-occurring anxiety and depression symptoms affect ADHD symptom–cognition relationships differently in middle- and older-age cohorts. Based on previous evidence of decrease in ADHD prevalence with age we expected participants in the older cohort to report fewer ADHD symptoms than middle-aged participants. Guided by our previous work on the middle-aged adults [52] we expected cognitive performance to be predominantly associated with symptoms of inattention in older adults.

## Methods

### Participants

The study sample was drawn from the PATH Through Life Project, a large longitudinal study of mental health and ageing in participants across three age groups (20–24, 40–44, 60–64 years at baseline) with a four-yearly follow-up ‘waves’ of assessment for up to 20 years [53]. The baseline sample comprised of individuals selected randomly from the electoral roll from the city of Canberra and the adjacent town of Queanbeyan, Australia (which provides a representative population sample because enrolment to vote is a legal requirement for adult Australian citizens).

The present study used data from the third wave of assessment (the ASRS was introduced in this wave) of the MA (40–44 years at baseline) and OA (60–64 years at baseline) cohorts. There were 2182 participants in MA cohort aged 48–52 years (mean age 50.7±1.5 years) and 1973 individuals in the OA cohort aged 68–72 years (mean age 70.6±1.5 years). Twenty-six (1%) and fifty-three participants (2.8%) in the MA and OA cohorts respectively did not complete the ASRS questionnaire and were excluded from further analysis. Being older was associated with increased odds of not completing the questionnaire in the OA cohort. However, non-responders did not differ significantly (*p*>0.05) from responders with respect to gender, total years of education and the levels of anxiety or depression symptoms in either cohort. Participants meeting at least one of the following criteria – history of epilepsy, brain tumour/infection and stroke and with missing data were excluded. Mini Mental State Examination (MMSE) was included in the assessment of the OA cohort to screen for probable dementia. Participants with scores of 23 or less (which is indicative of probable dementia [56]) were also excluded from the analyses. MA adults were not assessed using MMSE since this test was not designed to provide cognitive data on young or middle-aged adults and only presents a challenge for older individuals with significant cognitive difficulties. One MA participant reported taking medically prescribed dexamphetamine (which is used in ADHD treatment) and was excluded since information on dosage, duration of treatment and side effects (such as insomnia and increased anxiety and irritability) was not available, which could potentially affect cognitive performance. A final sample of n = 3443 (50% male) was available for this investigation.

### Procedure

Written informed consent for participation in the PATH project was obtained from all participants according to the ‘National Statement’ guidelines of the National Health and Medical Research Council of Australia and following a protocol approved by the Human Research Ethics Committee of The Australian National University. For wave 3 of the PATH study, on which the present analysis is based, all participants were assessed by trained interviewers to be capable of providing informed consent.

Participants were surveyed once every four years for information on physical and mental health, lifestyle and social factors (for details see [53]). Cognitive assessments were a part of the interview conducted by trained interviewers. The interview lasted for approximately 2 hours although this varied between individuals and was somewhat longer in the older-age group. The assessment was designed to alternate between questionnaire items, psychometric measures, and anthropometrical measures so as to decrease the effects of fatigue and attention lapses. A tea break was also offered in the middle of the assessment. In addition, to minimize the effects of fatigue and attention on sensitive measures, task presenting most difficulty were performed early in the interview.

### Measures

The short form of the ASRS was used, which consists of a checklist of six questions regarding symptoms of ADHD based on the diagnostic criteria of DSM-IV [54], [55]. The items in the ASRS questionnaire are shown in Table S1 in File S1. Each item requires participants to rate how frequently they experienced a particular symptom of ADHD in the past six months on a five-point response scale from “never” [0] to “very often” [4]. A summary score (ASRS-6 score) with a possible range of 0–24 was obtained as an equally weighted sum of response scores for all questions. Higher scores indicate increased risk of ADHD. This screening tool has performed well in validation studies (sensitivity = 68.7% and specificity = 99.5%) and has high concordance with clinician diagnosis (area under the receiver operator curve of 0.90) [55]. Factor analysis of the ASRS reported previously [57] suggest that the screener is a two-dimensional measure rather than a single unitary measure. Items 1 to 4 relate to inattention symptoms and load on one factor (inattentiveness) and items 5 and 6 load on a second factor (hyperactivity) [57].

Depression and anxiety symptoms were assessed using the Patient Health Questionnaire (PHQ). This is a short version of the patient questionnaire component of the Primary Care Evaluation of Mental Disorders (PRIME-MD) instrument [58], [59]. We generated measures of depression and anxiety-related disorders from: 9 items related to depression symptoms [rated on a 4-point scale from “not at all” (1) to “nearly every day” (4)]; 7 items related to anxiety symptoms [rated on a 3-point scale from “not at all” (1) to “more than half the days” (3)]; and 5 items related to panic disorder [rated on a 2-point scale of “no” (1) and “yes” (2)] following the coding algorithm provided in the PHQ instruction manual (available from Patient Health Questionnaire Screeners; http://www.phqscreeners.com/overview.aspx). Variables for panic disorder and other anxiety syndromes were combined to generate a binary categorical variable for anxiety symptoms [both panic disorder and other anxiety syndrome absent (0), either panic disorder or other anxiety syndrome present (1)].

### Cognitive Tests

Cognitive data were collected from participants as described previously [52]. The cognitive performance measures used in this study include: the Spot-the-Word Test (STW) which is a measure of verbal ability [60]; the Trail Making Tests A and B (TMT-A and TMT-B) for visual attention and task-switching [61], [62]; the Symbol-Digit Modalities Test (SMDT) for information processing speed and attention [63]; immediate and delayed recall of the first trial of the California Verbal Learning Test [64]; the Digits Span Backwards (DSB) task from the Wechsler Memory Scale for verbal working memory [65]; and Simple and Choice Reaction Time (SRT and CRT) [66]. Further details of the cognitive tests are provided in Table S2 in File S1. Percentage of missing data for the cognitive tests were as follows: Spot-the-Word Test (4.2%), Trail Making Test (Part A) (2.6%), Trail Making Test (Part B) (2.9%), Symbol-Digit Modalities Test (2.6%), Immediate Recall (1.7%), Delayed Recall (1.8%), Digits Span Backwards (2.2%), Simple Reaction Time (4.7%) and Choice Reaction Time (4.7%).

### Statistical Analysis

All statistical analyses were conducted using SPSS 18 and Amos version 20 (Chicago: SPSS Inc.). Missing data for cognitive measures were imputed using Expectation-Maximization method in SPSS. Means and standard deviations were computed for the ASRS-6 and cognitive test scores. As recommended [54], [55], participants were grouped into four strata based on ASRS-6 with the following score ranges: 0–9 (stratum I), 10–13 (stratum II), 14–17 (stratum III) and 18–24 (stratum IV). Comparisons between MA and OA were performed using Student’s t-tests and Pearson’s Chi-square tests for the continuous and categorical ASRS measures respectively (Table 1). For each cognitive test z-scores were generated. Higher scores on TMT and RT tasks indicate a worse performance, while a higher score on all other tests indicate better cognitive function. Pearson correlations were computed for the ASRS latent factors and cognitive test scores (Tables S3 and S4 in File S1).

**Table 1:** Socio-demographic characteristics and measures of ADHD, anxiety and depression symptom and cognitive performance in the sample.

		MA (n = 1907)		OA (n = 1536)				
		Mean (SD)/freq	Std. Res	Mean (SD)/freq	Std. Res	t/χ^2^	df	*p*
Gender						7.365	1	0.007
	Males	907 (47.6%)	−1.3	802 (52.2%)	1.4			
	Females	1000 (52.4%)	1.3	734 (47.8%)	1.4			
Age (years)		50.7 (1.5)		70.6 (1.5)		−386.499	3441	<0.001
Education (years)		14.9 (2.2)		14.1 (2.6)		9.690	3441	<0.001
ASRS-6 score		8.15 (3.40)		6.80 (3.25)		11.824	3441	<0.001
ASRS strata						89.324	3	<0.001
	I	1291 (67.7%)	−3.1	1251 (81.4%)	3.5			
	II	499 (26.2%)	4.1	251 (16.3%)	−4.6			
	III	99 (5.2%)	3.3	29 (1.9%)	−3.7			
	IV	18 (0.9)%	1.5	5 (0.3%)	−1.6			
ASRS score categories						31.203	1	<0.001
	0–13	1790 (93.9%)	−0.8	1502 (97.8%)	0.9			
	14–24	117 (6.1%)	3.6	34 (2.2%)	−4.1			
Depression symptoms present						24.620	1	<0.001
	Yes	258 (13.5%)	3.1	126 (8.2%)	−3.5			
	No	1644 (86.2%)	−1.1	1410 (91.8%)	1.2			
Anxiety symptoms present						19.227	1	<0.001
	Yes	106 (5.6%)	2.9	39 (2.5%)	−3.2			
	No	1801 (94.4%)	−0.6	1497 (97.5%)	0.7			
Spot-the-Word Test^a^		51.77 (4.98)		53.27 (4.92)		−8.800	3441	<0.001
Trail Making Test-A (seconds)^b^		25.78 (7.35)		35.55 (12.34)		−28.813	3441	<0.001
Trail Making Test-B (seconds)^b^		57.40 (20.13)		82.57 (31.83)		−28.230	3441	<0.001
Simple Reaction Time (seconds)^b^		0.24 (0.05)		0.28 (0.07)		−21.618	3441	<0.001
Choice Reaction Time (seconds)^b^		0.30 (0.04)		0.34 (0.05)		−28.206	3441	<0.001
Immediate Recall^a^		8.18 (2.17)		6.70 (2.21)		19.637	3441	<0.001
Delayed Recall^a^		7.38 (2.37)		5.98 (2.32)		17.401	3441	<0.001
Symbol-Digit Modalities Test^a^		59.73 (8.54)		48.58 (8.79)		37.595	3441	<0.001
Digit Span Backwards^a^		5.75 (2.29)		5.17 (2.17)		7.578	3441	<0.001

MA: middle-age cohort; OA: older-age cohort; ASRS: adult ADHD Self-Report Scale; freq: frequency; Std. Res: standardised residuals.

a number of correct responses;

b time taken to complete.

We used multi-group structural equation modelling to test cross-group invariance of the ASRS factor structure (Aim 1) and compare ADHD symptom–cognition relationships in MA and OA groups (Aim 2). First, cross-group invariance of the ASRS factor structure was tested using latent factor SEM in the framework of confirmatory factor analysis [67]. Equivalence of ASRS across cohorts was examined according to the procedure outlined in [67]. Once the ASRS factor structures for both cohorts were determined, a second set of SEMs was generated, which included the ASRS latent factors, the cognition variables and covariates to investigate ADHD symptom–cognition relationships. SEMs were fitted using maximum likelihood methods. Standard errors of the regression estimates were calculated from 2000 bootstrap-resampled datasets. Model fit was assessed using goodness-of-fit indices such as tests for chi-square distribution and root-mean square error of approximation (RMSEA; optimal value <0.05 [68]); adjusted goodness of fit index (AGFI; optimal value >0.9 [69]); comparative fit index (CFI; optimal value >0.9 [70]); Akaike information criterion (AIC; [71]); Browne-Cudeck criterion (BCC; [72]). Models with the lowest values for AIC and BCC are considered to have the best fit to the data. When sample sizes are large, as in this study, chi-square statistics can be significant even when the differences between the models being compared are small. We therefore also used a range of other goodness-of-fit indices to obtain a broader assessment of model fit.

Analysis of ASRS factor structure: Previous factor analysis has established that ASRS measures two correlated constructs, Inattention and Hyperactivity [57]. We treated these as latent factors in the SEMs, designated as *Inatt* (ASRS Items 1–4, Table S1 in File S1) and *Hyperact* (ASRS Items 5 and 6, Table S1 in File S1), respectively. We first tested the validity of the reported two-factor structure of the ASRS for the MA and OA cohorts individually (Table 2; Models MA and OA). We then examined the equivalence of the two-factor structure in both cohorts simultaneously through a series of models (Table 2; Models 1–6) with different parameters constrained. Model 1 was the baseline model where all parameters were allowed to vary between the two cohorts. For the other models the followings constraints were applied: Model 2– factor loadings (FL); Model 3– FL, variance of both factors (FV) and covariance between factors (FC); Model 4– FL and FC; Model 5a – FL, FC and variance of *Inatt* (V_Inatt_); Model 5b – FL, FC and variance of *Hyperact* (V_Hyperact_); and Model 6: FL, FC and residual variance (RV) of the ASRS items. Since the more constrained models were nested within the initial model, the Likelihood Ratio Test (difference in Chi-square statistics relative to the change in degrees of freedom) along with other goodness-of-fit indices (see above) was used to select the more parsimonious model.

**Table 2:** Goodness-of-fit statistics from single cohorts (MA, OA) and multi-group (MA+OA) SEMs assessing the ASRS factor structure.

Model	Constraints	?^2^	*df*	RMSEA	AGFI	CFI	AIC	BCC	Δχ^2^	Δ*df*	Δ*p*
*Individual group analyses*											
MA	−	46.386	9	0.047	0.981	0.984	70.386	70.474	−	−	−
OA	−	68.586	9	0.065	0.965	0.958	92.586	92.695	−	−	−
*Multi-group analyses*											
1	Nil	111.325	18	0.039	0.974	0.975	159.235	159.524	−	−	−
2	FL	112.065	22	0.034	0.979	0.976	152.065	152.231	0.740^a^	4	0.946
3	FL+FV+FC	131.554	25	0.035	0.978	0.972	165.554	165.695	19.489^b^	3	<0.001
4	FL+FC	113.609	23	0.034	0.980	0.976	151.609	151.767	1.544^b^	1	0.214
5a	FL+FC+V_Inatt_	125.063	24	0.035	0.978	0.973	161.063	161.212	11.454^c^	1	<0.001
5b	FL+FC+V_Hyperact_	119.023	24	0.034	0.979	0.975	155.023	155.172	5.414^c^	1	0.020
6	FL+FC+RV	158.245	28	0.037	0.976	0.966	186.245	186.361	44.636^c^	5	<0.001

MA: middle-age cohort; OA: older-age cohort; FL: Factor loadings of ASRS items on *Inatt* and *Hyperact* latent factors; FV: Factor variance for *Inatt* and *Hyperact*; FC: Covariance between *Inatt* and *Hyperact*; V_Inatt_: Variance of *Inatt*; V_Hyperact_: Variance of *Hyperact*; RV: Residual Variance of ASRS items.

RMSEA: root-mean square error of approximation (optimal value <0.05); AGFI: adjusted goodness of fit index (optimal value >0.9); CFI: comparative fit index (optimal value >0.9); AIC: Akaike information criterion; BCC: Browne-Cudeck criterion. Models with the lowest values for AIC and BCC are considered to have the best fit to the data.

a compared to Model 1;

b compared to Model 2;

c compared to Model 4.

**Figure 1:**
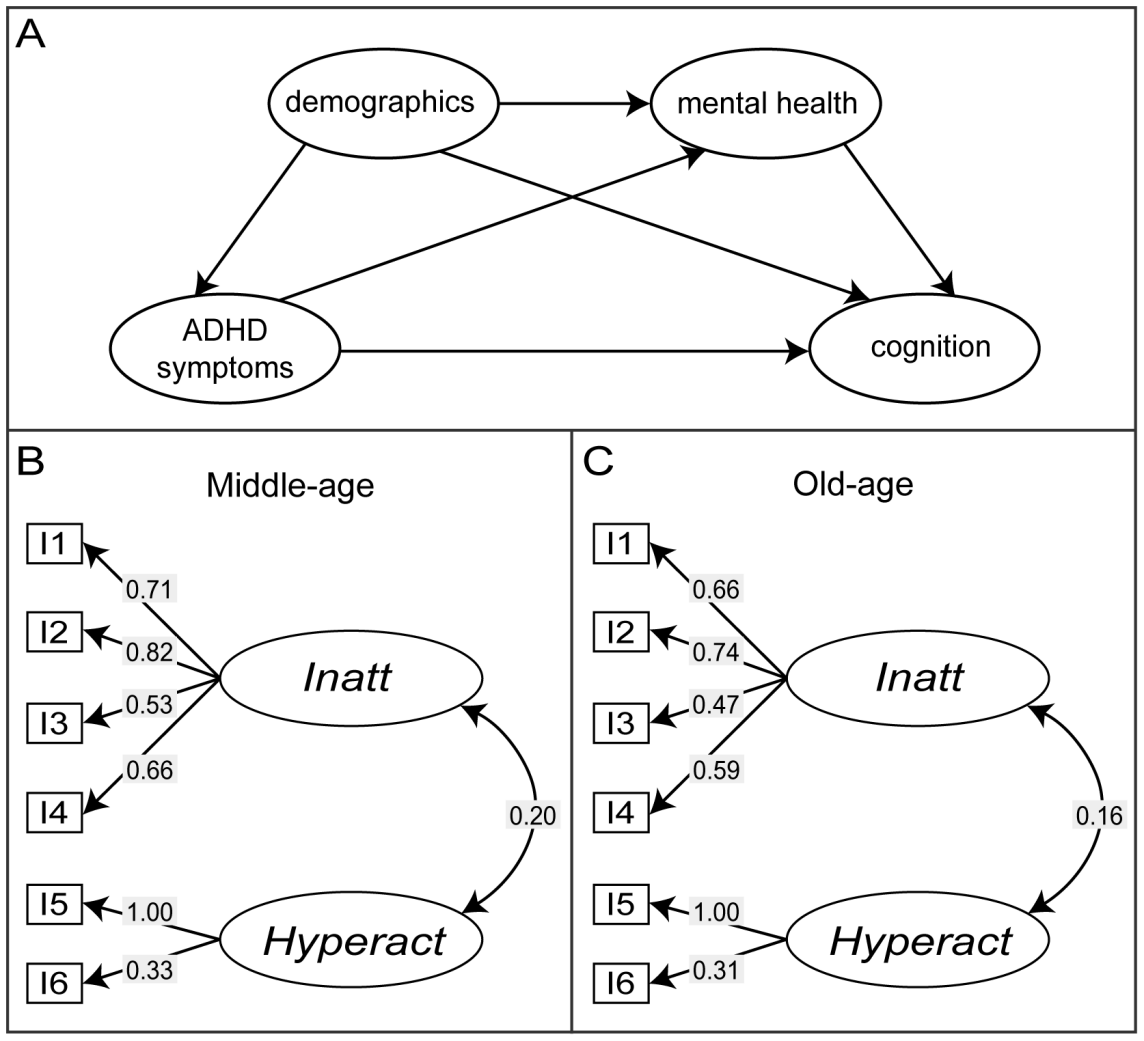
Theoretical model for ADHD symptom–cognition relationships and the factor structure of the ASRS. A) The theoretical model for ADHD symptom–cognition relationships examined in multi-group SEM analysis. Demographic variables include gender, age and education. Anxiety and depression symptom measures are indicated as the mental health variable in the diagram. B) and C) Models representing the ASRS factor structure in MA cohort (B) and OA cohort (C). Standardised factor loadings and covariance between the latent factors – *Inatt* and *Hyperact* are shown. Arrows reflect direction of relationships between variables.

Analysis of ADHD symptom–cognition relationships: SEMs generated for assessing the effect of ASRS latent factors on cognitive performance included cognitive test scores as dependent variables and *Inatt*, *Hyperact*, sociodemographic variables and mood-disorder symptoms as predictors. A schematic representation of our theoretical model is shown in Figure 1A. Factor loadings of the ASRS items on *Inatt* and *Hyperact* and covariance between the factors were constrained to be equal across both groups. All other parameters were estimated freely. To avoid multi-collinearity we used separate models for cognitive variables (Immediate Recall vs. Delayed Recall and Simple Reaction Time vs. Choice Reaction Time) that were highly correlated (*r*>0.7; Tables S3 and S4 in File S1). Model refinement was guided by comparison of goodness of fit statistics as described above, with non-significant paths (*p*>0.1) removed to improve the overall parsimony of the models. Only the final, most-parsimonious model and the coefficients of significant paths are reported. Results were considered significant at *p*≤0.05.

We also analysed the factor structure of the cognitive tests used in the study. The factor structure that represents the best fit to both cohorts are shown in Figure S1. We generated SEM models with the cognition latent factors as dependent variables using the method outlined above. These results are presented as Table S5 in File S1 and Figure S2.

## Results

Demographic characteristics, symptom measures for ADHD, depression and anxiety, and cognitive test scores for both cohorts are presented in Table 1 (Information about access to data is available at: http://crahw.anu.edu.au/research/projects/personality-total-health-path-through-life/data ). The OA cohort reported significantly lower levels for ADHD, depression and anxiety. For the ASRS, 2.2% of participants in the OA cohort scored 14 or above, which has previously been identified as indicative of ADHD diagnosis [55], compared with 6.1% in the MA cohort. The OA cohort also has fewer individuals classified in the II, III and IV ASRS strata than the MA cohort. OA participants performed significantly worse than MA participants in all cognitive tests with the exception of the Spot-the-Word Test of verbal ability.

### Equivalence of ASRS Instrument in MA and OA Cohorts

The data are well described by models incorporating the proposed two-factor (*Inatt*+*Hyperact)* ASRS structure in both cohorts individually (Table 2, Models MA and OA). Factor loadings and factor covariance are presented in Figure 1 for MA (panel B) and OA (panel C) adults. The covariance between the latent factors – *Inatt* and *Hyperact* was lower in the OA adults compared to MA adults. The two-factor model also had acceptable goodness-of-fit indices (Table 2) when both cohorts were combined (Table 2: Model 1). When FLs for the OA cohort were constrained to be equal to those estimated for MA adults, the fit indices changed only slightly (Table 2: Model 2). For an increase in 4 degrees of freedom in Model 2 compared to Model 1 the corresponding change in χ^2^ was not significant at an alpha level of 0.05. Constraining FLs, FVs and FC to be the same across groups (Table 2: Model 3) also resulted in a well-fitting model. However, the Likelihood Ratio Test indicated that this model was significantly different from Model 2, suggesting that the variance and covariance of the latent factors differed significantly between the age cohorts. To examine this further, we constrained only FC (Table 2: Model 4) and found the model not to be significantly different from Model 2, which suggests that while the covariance between *Inatt* and *Hyperact* was lower in the OA cohort it was not significantly different from the MA cohort. Adding further constraints on the variances of either *Inatt* or *Hyperact* (Table 2: Model 5a and b) or residual variances of the ASRS items (RVs; Table 2: Model 6) caused significant changes in model fit compared to Model 4. This indicates that both the variance of the latent factors and residual variances of the ASRS items differed significantly between the age cohorts. Thus, Model 4 where FLs and FC were constrained to have the same values across cohorts exhibited the best fit to the data for both age groups and hence, only this model was used for subsequent analyses.

### Effect of ADHD Symptoms on Cognitive Performance

**Figure 2:**
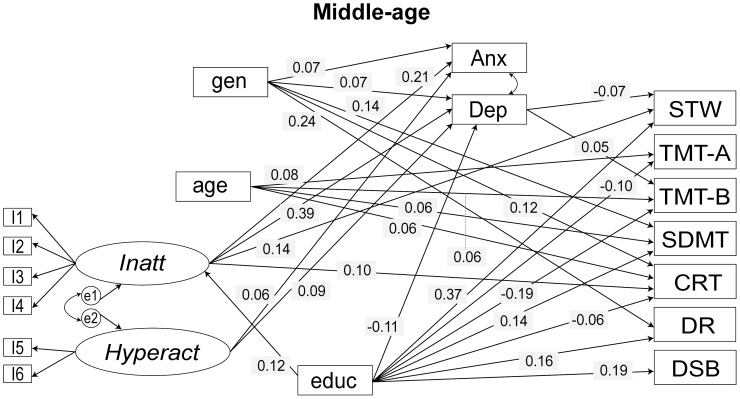
Final model for ADHD symptom–cognition analyses for MA cohort. Only paths significant at *p*<0.01 are shown. Arrows reflect direction of relationships between variables. Standardised regression coefficients are shown. Factor loadings and covariance between the latent factors – *Inatt* and *Hyperact*, which were constrained equal across groups in the analyses, and correlation between cognitive test measures are not shown for the sake of simplicity. I: item; gen: gender; educ: education; *Inatt*: latent factor Inattention; *Hyperact*: latent factor Hyperactivity; DEP: depression symptom measure; ANX: anxiety symptom measure; STW: Spot-the-Word Test; TMT-A: Trail Making Test A; TMT-B: Trail Making Test B; SDMT: Symbol-Digit Modalities Test; CRT: Choice Reaction Time; DR: Delayed Recall; DSB: Digit Span Backwards.

**Figure 3:**
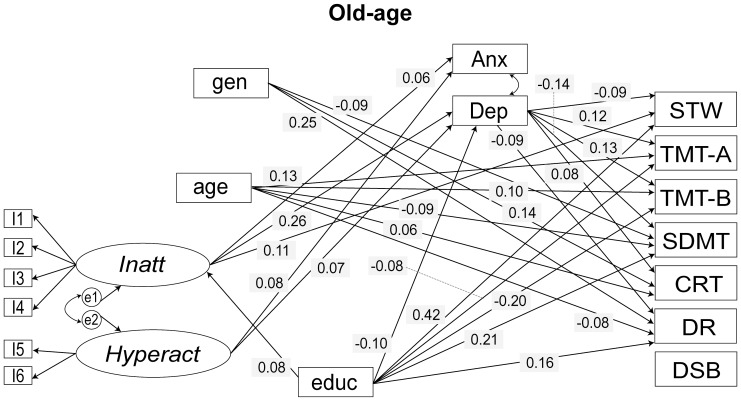
Final model for ADHD symptom–cognition analyses for OA cohort. Only paths significant at *p*<0.01 are shown. Arrows reflect direction of relationships between variables. Standardised regression coefficients are shown. Factor loadings and covariance between the latent factors – *Inatt* and *Hyperact*, which were constrained equal across groups in the analyses, and correlation between cognitive test measures are not shown for the sake of simplicity. I: item; gen: gender; educ: education; *Inatt*: latent factor Inattention; *Hyperact*: latent factor Hyperactivity; DEP: depression symptom measure; ANX: anxiety symptom measure; STW: Spot-the-Word Test; TMT-A: Trail Making Test A; TMT-B: Trail Making Test B; SDMT: Symbol-Digit Modalities Test; CRT: Choice Reaction Time; DR: Delayed Recall; DSB: Digit Span Backwards.

Using multi-group SEMs we investigated the effects of ADHD symptoms on cognitive performance, while controlling for sociodemographic variables and mood disorder symptoms (Figure 1A). The ASRS two-factor model (Model 4) described above was included as a measure of ADHD symptoms. The cognitive outcome variables were – Spot-the-Word Test, Trail Making Test, Symbol-Digit Modalities Test, Choice Reaction Time, Delayed Recall and Digits Span Backwards. The baseline model evaluated the theoretical model shown in Figure 1A and had acceptable model fit indices: χ^2^ = 625.265, *df* = 125, *p*<0.001; RMSEA = 0.034; AGFI = 0.947; CFI = 0.955; AIC = 1061.265; BCC = 1066.173. However, coefficients for several paths in the model were not significant. Hence, regression paths with *p*>0.1 were removed to give a final model with improved overall fit to the data (χ^2^ = 682.882, *df* = 189, *p*<0.001; RMSEA = 0.028; AGFI = 0.961; CFI = 0.956; AIC = 989.882; BCC = 992.342). Significant paths (*p*≤0.01) in the final model are shown in Figures 2 and 3 and regression coefficients are presented in Table 3. Models with Immediate-recall and Simple Reaction Time are not reported since they were not significantly associated with the ADHD latent variables in either cohort.

**Table 3:** Path coefficients from multi-group SEM analyses of ADHD symptom–cognition relationships.

			MA			OA		
			Coefficients§	SE†	*p*	Coefficients§	SE†	*p*
age	→	*Hyperact*	−	−	−	−0.034	0.016	0.032
gender	→	*Hyperact*	−	−	−	0.093	0.049	0.056
gender	→	*Inatt*	−0.062	0.032	0.046	−	−	−
education	→	*Inatt*	0.035	0.007	<0.001	0.018	0.007	0.003
gender	→	anxiety	0.031	0.010	0.002	−	−	−
education	→	anxiety	−0.004	0.003	0.080	−0.005	0.002	<0.001
*Inatt*	→	anxiety	0.077	0.014	<0.001	0.017	0.011	0.056
gender	→	depression	0.565	0.169	0.001	−	−	−
education	→	depression	−0.205	0.040	<0.001	−0.114	0.028	<0.001
*Inatt*	→	depression	2.463	0.215	<0.001	1.300	0.172	<0.001
education	→	STW	0.154	0.009	<0.001	0.153	0.009	<0.001
*Inatt*	→	STW	0.198	0.034	<0.001	0.185	0.045	<0.001
depression	→	STW	−0.015	0.005	0.002	−0.030	0.008	<0.001
age	→	TMT-A	0.047	0.013	<0.001	0.060	0.011	<0.001
gender	→	TMT-A	−0.061	0.035	0.085	−0.125	0.032	<0.001
education	→	TMT-A	−0.038	0.009	<0.001	−0.020	0.007	<0.001
*Inatt*	→	TMT-A	0.062	0.034	0.069	−	−	−
depression	→	TMT-A	0.011	0.005	0.024	0.029	0.007	<0.001
age	→	TMT-B	0.033	0.012	0.006	0.055	0.014	<0.001
education	→	TMT-B	−0.074	0.010	<0.001	−0.064	0.008	<0.001
*Hyperact*	→	TMT-B	−	−	−	−0.035	0.017	0.034
depression	→	TMT-B	0.012	0.005	0.006	0.039	0.008	<0.001
age	→	SDMT	−0.040	0.013	0.002	−0.053	0.015	<0.001
gender	→	SDMT	0.258	0.037	<0.001	0.170	0.037	<0.001
education	→	SDMT	0.060	0.010	<0.001	0.075	0.009	<0.001
anxiety	→	SDMT	−0.147	0.069	0.063	−	−	−
depression	→	SDMT	−	−	−	−0.045	0.009	<0.001
age	→	CRT	0.028	0.010	0.006	0.036	0.014	0.010
gender	→	CRT	0.172	0.030	<0.001	0.234	0.041	<0.001
education	→	CRT	−0.018	0.008	0.011	−	−	−
*Inatt*	→	CRT	0.107	0.028	<0.001	−	−	−
depression	→	CRT	−	−	−	0.024	0.008	0.001
age	→	DR	−	−	−	−0.054	0.016	<0.001
gender	→	DR	0.475	0.042	<0.001	0.499	0.047	<0.001
education	→	DR	0.069	0.010	<0.001	0.062	0.009	<0.001
*Inatt*	→	DR	0.079	0.042	0.059	−	−	−
*Hyperact*	→	DR	0.045	0.021	0.027	−	−	−
anxiety	→	DR	−0.213	0.112	0.046	−	−	−
depression	→	DR	−0.015	0.007	0.019	−0.031	0.008	<0.001
education	→	DSB	0.084	0.010	<0.001	−	−	−

Coefficients for ASRS items, which were same for both cohorts are not shown.

Only paths significant at *p*<0.1 are shown.

§ unstandardised estimate.

† standard errors were computed from 2000 bootstrap-resampled datasets.

MA: middle-age cohort; OA: older-age cohort; *Inatt*: latent factor Inattention; *Hyperact*: latent factor Hyperactivity; STW: Spot-the-Word test; TMT-A: Trail Making Test A; TMT-B: Trail Making Test B; SDMT: Symbol-Digit Modalities Test; CRT: Choice Reaction Time; DR: Delayed Recall; DSB: Digit Span Backwards.

*Inatt* and *Hyperact* have limited direct effects on cognitive performance. The most notable was a positive association, observed in both cohorts, between *Inatt* and Spot-the-Word Test, implying that higher levels of inattention symptoms are associated with greater verbal intelligence. *Inatt* was also positively associated with Choice Reaction Time, i.e., with slower reaction time in the MA cohort. *Hyperact* was positively associated with the Delayed Recall test (i.e. poor performance) on the MA cohort and Trail Making Test (Part B) (i.e. better performance) in the OA cohort. *Inatt* was associated with depression and anxiety symptoms more strongly in the MA than the OA cohort.

We also investigated the association between ADHD symptoms and latent variables obtained from factor analysis of the cognitive test scores. Factor analysis identified four latent factors (Figure S1) broadly representing: working memory, information processing speed, speed/executive function and verbal memory. The most parsimonious model obtained from the SEM analysis with the cognition latent factors as dependent variables fit the data well for both cohorts (Table S5 in File S1 and Figure S2) and was very similar to the model described in Table 3 and Figures 2 and 3. It is important to note that the cognition tests were included in the PATH study only as indicators of different kinds of cognitive ability and were not designed to measure latent constructs. Hence, cognitive domains represented by these latent factors are both difficult to interpret and would not be considered stable as they have less than three indicators.

### Group Differences in Sociodemographic Variables

The effect of the covariates was similar in the two cohorts, but there were some notable differences. Gender was strongly associated with mood variables and marginally with *Inatt* only in the MA cohort. It had an effect on the Trail Making Test (Part A) in the OA cohort and had no effect on any of the cognitive variables in the MA cohort. Effects of age on the cognitive variables were similar across both cohorts although the effects were stronger in the OA cohort. A point of contrast was the significant effect of age on Delayed Recall test on OA cohort that was absent in the MA cohort. Age was marginally associated with *Hyperact* in older adults only. Education had negative effects on mood variables in both cohorts although the effect on anxiety was much stronger in the OA cohort. The effect of education on *Inatt* was positive in both cohorts and comparatively stronger in the MA cohort, implying that more education is associated with higher levels of inattention. Greater levels of depression symptoms were associated with poorer cognitive performance generally, but the effect is much stronger in the OA cohort. These results did not differ significantly when latent factors underlying the cognition tests were included as dependent variables in the SEM models (Table S5 in File S1 and Figure S2).

## Discussion

In this study we investigated ADHD symptoms in a large population-based sample of older adults, focussing on their effects on cognition. Importantly, we established that the underlying construct of the ADHD screener, ASRS, which has been validated in young adults, is similar in middle- and older-aged adults. We could therefore be confident that results obtained using the ASRS related to the same underlying psychological construct in both age groups.

Significant findings from the study are: i) older adults reported fewer/less severe ADHD symptoms compared to middle-aged adults; ii) the effect of ADHD symptoms on cognitive performance was weaker in older adults compared to middle-aged adults; iii) in OA adults the ADHD symptom–cognition relationship was mostly indirect and mediated through the strong association between depression symptoms and cognition.

We found that 2.2% of the OA cohort met the suggested cut-off for the ASRS-6 score that has been linked to clinical diagnosis in previous studies. This estimate falls within the range of 1–2.5% ADHD prevalence reported in the literature for older adults [26], [27], and is substantially lower than the estimate for the MA cohort (6.2%). A previous study [27] had found that older elderly adults (71–94 years) reported fewer ADHD symptoms compared to younger elderly adults (60–70 years). Hence, our study replicated previous findings indicating that, when applying the criteria developed for young adults, the prevalence of ADHD symptoms appears to decrease with age. It is important to note that in DSM-5 the symptom threshold for diagnosis of ADHD in adults has been reduced. The impact of this change on the prevalence of ADHD in this age group remains to be investigated.

There may be several reasons for this apparent decline in ADHD symptom levels in old age. The effects of ADHD may become less discernable from other general age-related changes. Thus, even though, as we have demonstrated here, the ASRS appears to measure variation in the same psychological construct in old age, it may do so with lower sensitivity. The relative frequency of people with ADHD in the population will also tend to decline in old age because they have a relatively lower life expectancy [27]. Their relative participation rate may also decline due to the accumulating effects of greater rates of accidents, substance abuse and mood disorders [73], [74].

We did not find any significant gender difference in ADHD symptoms in older adults. This result is consistent with our previous observation [75] and that of de Zwaan et al. (2012) [76], but contrary to a previous reports that found significantly higher prevalence of ADHD in males [77]. It is known that gender difference in ADHD prevalence is more extreme in paediatric samples compared to adolescent and adult samples. Since childhood referrals are initiated by parents and teachers, girls without the hyperactivity component of ADHD are less likely to be clinically diagnosed [78]. In contrast, women experience more internalizing problems than men [79], which may lead to higher rates of self-referrals in adulthood. Our results, which are obtained from a general population sample and are free from clinical ascertainment bias, suggest that the relative increase in female ADHD prevalence from childhood to adulthood is not a result of relative over-diagnosis of women.

We replicated the factor structure of the ASRS reported by Hesse et al. (2013) [57] in both MA and OA cohorts. Our results suggest that the ASRS measures similar constructs in both age groups. The latent factors – *Inatt* and *Hyperact* were more strongly correlated in MA than OA adults, however, this difference was not statistically significant. In a previous study of two samples of different age [57], correlation between the latent factors was lower in the sample with a mean age of 35.5 years than the relatively younger sample (mean age 24.6 years). Whether this difference was statistically significant has not been reported. Studies in young and old adults also suggest that among the different ADHD subtypes, the frequency of the combined subtype relative to the other subtypes declines with age [27], [80]. Thus, with age, symptoms of inattention and hyperactivity appear both to decrease and to occur less frequently in combination.

In both MA and OA cohorts the Spot-the-Word Test, which measures verbal ability, was positively associated with symptoms of inattention, an effect that we previously reported for the MA cohort [52]. The sizes of the effects were similar and remained significant after controlling for depression symptoms, which were negatively associated with the Spot-the-Word Test, in both cohorts. One possible explanation is suggested by a previous study that found that while ADHD adults performed relatively poorly on verbal learning tests, they did not lose information at a greater rate once it was encoded [81]. In older adults, reaction time was not associated with *Inatt*, in contrast with the MA cohort, suggesting that information processing speed is less affected by symptoms of inattention in older adults.

Surprisingly, in the OA cohort, greater hyperactivity was associated with better task-switching abilities as measured by the Trail Making Test (Part B). However, this association did not reach the more stringent significance threshold of *p*<0.01. This might suggest that hyperactivity symptoms in this age-group are beneficial for certain cognitive activities. It is possible that hyperactivity symptoms in ageing brains make the system noisier at the neuronal level. While noise is generally considered detrimental, under certain circumstances it may aid in processing of weak stimuli through the phenomenon of stochastic resonance [82]. The motor and sensory input associated with hyperactivity could increase noise thus enabling neuronal responses that otherwise would not occur and resulting in improved responses to certain tasks. Such improvements are more likely in older brains since ageing associated neurobiological decline leads to weaker stimuli. The positive association between hyperactivity and cognitive performance in older adults is in contrast to previous reports in younger adults, where ADHD symptoms were related to worse cognitive outcomes [83]. It is important to note that the level of hyperactivity in the OA cohort is much less than that present in younger samples and that the ASRS contains only two items to detect hyperactivity symptoms. Hence, the positive association between symptoms of hyperactivity and task-switching ability in older adults needs to be investigated in future studies.

Our results suggest that the effects of ADHD symptoms are specific to particular cognitive domains, in contrast to a previous study by Biederman et al (2011) [50] that reported consistent poor performance across a neuropsychological battery (assessing executive functions, learning and memory) in non-medicated ADHD patients. This difference may reflect important differences between the two studies. The previous study [50] was based on referred ADHD patients, who were on average younger than our sample. It reported cognitive effects of ADHD diagnosis and did not include comorbid mood disorders. Participants in our study were recruited randomly from the community and were not selected for ADHD. We investigated the effects of the full range of ADHD symptom levels rather clinical diagnosis and we differentiated between symptoms of inattention and hyperactivity and made adjustments for symptoms of anxiety and depression, which are common comorbidities of ADHD.

Our results suggest that inattention symptoms affect cognitive performance indirectly through their effect on depression in the OA cohort. *Inatt* was strongly associated with depression symptoms in both cohorts, and depression symptoms predict worse performance in all cognitive tests in the OA cohort but not in the MA cohort. We also found evidence of statistical mediation of *Inatt* on Symbol-Digit Modalities Test by depression in OA adults since the negative association between *Inatt* and Symbol-Digit Modalities Test became non-significant when depression symptoms were included in the model. Depression symptoms were also significantly associated with Symbol-Digit Modalities Test in OA adults. Thus, our results emphasise the importance of controlling for comorbid mood disorders when investigating ADHD symptom–cognition relationship, especially in older adults.

The strengths of this study are that it was conducted in a large representative population sample, and hence it is likely to be free of the biases that can be associated with clinical and convenience samples. Furthermore, the narrow age range of the sample removes the possible confounding effects of age within each cohort. Consequently, our results are likely to be generalisable to MA and OA populations. Our study included age groups that have not been well studied with respect to ADHD symptoms and thus is a significant addition to the growing literature in this area. We focus on cognitive performance and ADHD symptoms highlighting differences between middle- and old-age, which is important for understanding age-related cognitive change. Finally, we have modelled both direct ADHD symptom-cognition relationships and indirect effects mediated by symptoms of depression, demonstrating age-group differences in these direct and indirect pathways.

Limitations of the study include lack of clinical assessments for ADHD, depression and anxiety. In addition, symptom measures are based on self-reports, which may not be completely accurate (e.g. social desirability and current emotional state could introduce biases [84], [85]). However, the assessment instruments we used have been shown to have good sensitivity and specificity and they have been used in a number of previous studies and validated in different cultural settings [55], [58], [86]–[88]. Furthermore, we have included appropriate statistical evaluation to ensure that the ASRS measure is equivalent in MA and OA cohorts. A small number of cognitive tests have been analysed in the study. These tests were included in the PATH study as indicators of different abilities and were not designed to measure latent constructs. Hence, detailed analysis on the effect of ADHD symptoms on specific cognitive domains was not possible. Although we found evidence of statistical mediation by symptoms of depression on ADHD symptom–cognition relationships, due to the cross-sectional nature of the study we could not ascertain the causal relation between symptoms of ADHD and depression. Longitudinal follow-up of the cohort may contribute towards understanding the temporal relationships among these factors.

In conclusion, we have shown that although ADHD symptoms persist, they are less common in older compared to middle-aged adults. The relationship between ADHD symptoms and cognitive performance changes with age. While symptoms of depression are lower in older adults, they are much stronger predictors of cognitive performance and appear to mediate the effect of ADHD symptoms on cognition in this age group. Thus, better diagnosis and treatment of comorbid ADHD and mood disorders in older adults is required and might contribute to promoting cognitive health in late-life.

## Supporting Information

Figure S1 Models representing the latent structure of the cognitive tests used in the study in the MA (A) and OA (B) cohorts. Standardised factor loadings are shown. IR: Immediate Recall; DR: Delayed Recall; SRT: Simple Reaction Time; CRT: Choice Reaction Time; TMT-A: Trail Making Test A; TMT-B: Trail Making Test B; SDMT: Symbol-Digit Modalities Test; STW: Spot The Word test; DSB: Digit Span Backwards.
(TIF)

Figure S2 Final model for ADHD symptom–cognition analyses for MA (A) and OA (B) cohorts. Only paths significant at *p*<0.05 are shown. Arrows reflect direction of relationships between variables. Standardised regression coefficients are shown. gen: gender; educ: education; *Inatt*: latent factor Inattention; *Hyperact*: latent factor Hyperactivity; DEP: depression symptom measure; ANX: anxiety symptom measure. Indicators of the latent variables are not shown for the sake of clarity.
(TIF)

File S1 Tables S1–S5.
(DOCX)

## References

[1] Biederman J, Faraone SV (2005) Attention-deficit hyperactivity disorder. Lancet 366: 237–248 10.1016/S0140-6736(05)66915-2 16023516

[2] Karam RG, Bau CHD, Salgado CAI, Kalil KLS, Victor MM, et al (2009) Late-onset ADHD in adults: milder, but still dysfunctional. J Psychiatr Res 43: 697–701 10.1016/j.jpsychires.2008.10.001 19007940

[3] Barkley RA (2010) Attention Deficit Hyperactivity Disorder in Adults. Sudbury, Massachusetts: Jones & Bartlett Publishers.

[4] Berlin L, Bohlin G, Nyberg L, Janols L-O (2004) How well do measures of inhibition and other executive functions discriminate between children with ADHD and controls? Child Neuropsychol 10: 1–13 10.1076/chin.10.1.1.26243 14977511

[5] Biederman J, Monuteaux MC, Doyle AE, Seidman LJ, Wilens TE, et al (2004) Impact of executive function deficits and attention-deficit/hyperactivity disorder (ADHD) on academic outcomes in children. J Consult Clin Psychol 72: 757–766 10.1037/0022-006X.72.5.757 15482034

[6] Lambek R, Tannock R, Dalsgaard S, Trillingsgaard A, Damm D, et al (2011) Executive dysfunction in school-age children with ADHD. J Atten Disord 15: 646–655 10.1177/1087054710370935 20858784

[7] Seidman LJ (2006) Neuropsychological functioning in people with ADHD across the lifespan. Clin Psychol Rev 26: 466–485 10.1016/j.cpr.2006.01.004 16473440

[8] Adler LA (2010) Monitoring adults with ADHD: a focus on executive and behavioral function. J Clin Psychiatry 71: e18 10.4088/JCP.9066tx3c 20797374

[9] Nigg JT (2005) Neuropsychologic theory and findings in attention-deficit/hyperactivity disorder: the state of the field and salient challenges for the coming decade. Biol Psychiatry 57: 1424–1435 10.1016/j.biopsych.2004.11.011 15950017

[10] Bálint S, Czobor P, Komlósi S, Mészáros A, Simon V, et al (2009) Attention deficit hyperactivity disorder (ADHD): gender- and age-related differences in neurocognition. Psychol Med 39: 1337–1345 10.1017/S0033291708004236 18713489

[11] Rösler M, Casas M, Konofal E, Buitelaar J (2010) Attention deficit hyperactivity disorder in adults. World J Biol Psychiatry 11: 684–698 10.3109/15622975.2010.483249 20521876

[12] Kessler RC, Adler L, Barkley R, Biederman J, Conners CK, et al (2006) The prevalence and correlates of adult ADHD in the United States: results from the National Comorbidity Survey Replication. Am J Psychiatry 163: 716–723 10.1176/appi.ajp.163.4.716 16585449 PMC2859678

[13] Silva KL, Guimaraes-da-Silva PO, Grevet EH, Victor MM, Salgado CAI, et al.. (2012) Cognitive Deficits in Adults With ADHD Go Beyond Comorbidity Effects. J Atten Disord. doi:10.1177/1087054711434155. 10.1177/1087054711434155 22344317

[14] Swensen AR, Birnbaum HG, Secnik K, Marynchenko M, Greenberg P, et al (2003) Attention-deficit/hyperactivity disorder: increased costs for patients and their families. J Am Acad Child Adolesc Psychiatry 42: 1415–1423 10.1097/00004583-200312000-00008 14627876

[15] Secnik K, Swensen A, Lage MJ (2005) Comorbidities and costs of adult patients diagnosed with attention-deficit hyperactivity disorder. Pharmacoeconomics 23: 93–102 10.2165/00019053-200523010-00008 15693731

[16] Birnbaum HG, Kessler RC, Lowe SW, Secnik K, Greenberg PE, et al (2005) Costs of attention deficit-hyperactivity disorder (ADHD) in the US: excess costs of persons with ADHD and their family members in 2000. Curr Med Res Opin 21: 195–206 10.1185/030079904X20303 15801990

[17] Matza LS, Paramore C, Prasad M (2005) A review of the economic burden of ADHD. Cost Eff Resour Alloc 3: 5 10.1186/1478-7547-3-5 15946385 PMC1180839

[18] Bernfort L, Nordfeldt S, Persson J (2008) ADHD from a socio-economic perspective. Acta Paediatr 97: 239–245 10.1111/j.1651-2227.2007.00611.x 18254913

[19] Brod M, Schmitt E, Goodwin M, Hodgkins P, Niebler G (2012) ADHD burden of illness in older adults: a life course perspective. Qual Life Res 21: 795–799 10.1007/s11136-011-9981-9 21805205

[20] Polanczyk G, de Lima MS, Horta BL, Biederman J, Rohde LA (2007) The worldwide prevalence of ADHD: a systematic review and metaregression analysis. Am J Psychiatry 164: 942–948 10.1176/appi.ajp.164.6.942 17541055

[21] Polanczyk G, Rohde LA (2007) Epidemiology of attention-deficit/hyperactivity disorder across the lifespan. Curr Opin Psychiatry 20: 386–392 10.1097/YCO.0b013e3281568d7a 17551354

[22] Biederman J, Mick E, Faraone SV (2000) Age-dependent decline of symptoms of attention deficit hyperactivity disorder: impact of remission definition and symptom type. Am J Psychiatry 157: 816–818. 10784477 10.1176/appi.ajp.157.5.816

[23] Faraone SV, Biederman J, Spencer T, Mick E, Murray K, et al (2006) Diagnosing adult attention deficit hyperactivity disorder: are late onset and subthreshold diagnoses valid? Am J Psychiatry 163: 1720–1729 10.1176/appi.ajp.163.10.1720 17012682

[24] Fayyad J, De Graaf R, Kessler R, Alonso J, Angermeyer M, et al (2007) Cross-national prevalence and correlates of adult attention-deficit hyperactivity disorder. Br J Psychiatry 190: 402–409 10.1192/bjp.bp.106.034389 17470954

[25] Ebejer JL, Medland SE, van der Werf J, Gondro C, Henders AK, et al (2012) Attention deficit hyperactivity disorder in Australian adults: prevalence, persistence, conduct problems and disadvantage. PLoS ONE 7: e47404 10.1371/journal.pone.0047404 23071800 PMC3468512

[26] Kooij JJS, Buitelaar JK, Van Den Oord EJ, Furer JW, Rijnders CAT, et al (2005) Internal and external validity of attention-deficit hyperactivity disorder in a population-based sample of adults. Psychol Med 35: 817–827 10.1017/S003329170400337X 15997602

[27] Michielsen M, Semeijn E, Comijs HC, van de Ven P, Beekman ATF, et al.. (2012) Prevalence of attention-deficit hyperactivity disorder in older adults in The Netherlands. Br J Psychiatry. doi:10.1192/bjp.bp.111.101196. 10.1192/bjp.bp.111.101196 22878132

[28] Solanto MV, Wasserstein J, Marks DJ, Mitchell KJ (2012) Diagnosis of ADHD in Adults: What Is the Appropriate DSM-5 Symptom Threshold for Hyperactivity-Impulsivity? J Atten Disord 16: 631–634 10.1177/1087054711416910 21976031

[29] American Psychiatric Association (2013) Diagnostic and Statistical Manual of Mental Disorders. 5 ed. Washington, DC: American Psychiatric Publishing, Incorporated.

[30] Arcos-Burgos M, Acosta MT (2007) Tuning major gene variants conditioning human behavior: the anachronism of ADHD. Curr Opin Genet Dev 17: 234–238 10.1016/j.gde.2007.04.011 17467976

[31] Hoogman M, Rijpkema M, Janss L, Brunner H, Fernandez G, et al (2012) Current self-reported symptoms of attention deficit/hyperactivity disorder are associated with total brain volume in healthy adults. PLoS ONE 7: e31273 10.1371/journal.pone.0031273 22348063 PMC3277496

[32] Lubke GH, Hudziak JJ, Derks EM, van Bijsterveldt TCEM, Boomsma DI (2009) Maternal ratings of attention problems in ADHD: evidence for the existence of a continuum. J Am Acad Child Adolesc Psychiatry 48: 1085–1093 10.1097/CHI.0b013e3181ba3dbb 19797980 PMC2782551

[33] Nikolas MA, Burt SA (2010) Genetic and environmental influences on ADHD symptom dimensions of inattention and hyperactivity: a meta-analysis. J Abnorm Psychol 119: 1–17 10.1037/a0018010 20141238

[34] Shaw P, Gilliam M, Liverpool M, Weddle C, Malek M, et al (2011) Cortical development in typically developing children with symptoms of hyperactivity and impulsivity: support for a dimensional view of attention deficit hyperactivity disorder. Am J Psychiatry 168: 143–151 10.1176/appi.ajp.2010.10030385 21159727 PMC3268520

[35] Marcus DK, Norris AL, Coccaro EF (2012) The latent structure of attention deficit/hyperactivity disorder in an adult sample. J Psychiatr Res 46: 782–789 10.1016/j.jpsychires.2012.03.010 22480749 PMC3359405

[36] American Psychiatric Association (2000) Diagnostic and Statistical Manual of Mental Disorders. 4 ed. Washington, DC: American Psychiatric Publication.

[37] Fair DA, Nigg JT, Iyer S, Bathula D, Mills KL, et al (2012) Distinct neural signatures detected for ADHD subtypes after controlling for micro-movements in resting state functional connectivity MRI data. Front Syst Neurosci 6: 1–31 10.3389/fnsys.2012.00080 23382713 PMC3563110

[38] Carr L, Henderson J, Nigg JT (2010) Cognitive control and attentional selection in adolescents with ADHD versus ADD. J Clin Child Adolesc Psychol 39: 726–740 10.1080/15374416.2010.517168 21058121 PMC3059559

[39] Solanto MV, Schulz KP, Fan J, Tang CY, Newcorn JH (2009) Event-related FMRI of inhibitory control in the predominantly inattentive and combined subtypes of ADHD. J Neuroimaging 19: 205–212 10.1111/j.1552-6569.2008.00289.x 19594667 PMC2711513

[40] Hervey AS, Epstein JN, Curry JF (2004) Neuropsychology of adults with attention-deficit/hyperactivity disorder: a meta-analytic review. Neuropsychology 18: 485–503 10.1037/0894-4105.18.3.485 15291727

[41] Schoechlin C, Engel RR (2005) Neuropsychological performance in adult attention-deficit hyperactivity disorder: meta-analysis of empirical data. Arch Clin Neuropsychol 20: 727–744 10.1016/j.acn.2005.04.005 15953706

[42] McLean A, Dowson J, Toone B, Young S, Bazanis E, et al (2004) Characteristic neurocognitive profile associated with adult attention-deficit/hyperactivity disorder. Psychol Med 34: 681–692 10.1017/S0033291703001296 15099422

[43] Bridgett DJ, Walker ME (2006) Intellectual functioning in adults with ADHD: a meta-analytic examination of full scale IQ differences between adults with and without ADHD. Psychol Assess 18: 1–14 10.1037/1040-3590.18.1.1 16594807

[44] Epstein JN, Johnson DE, Varia IM, Conners CK (2001) Neuropsychological assessment of response inhibition in adults with ADHD. J Clin Exp Neuropsychol 23: 362–371 10.1076/jcen.23.3.362.1186 11404813

[45] Tucha L, Tucha O, Walitza S, Sontag TA, Laufkötter R, et al (2009) Vigilance and sustained attention in children and adults with ADHD. J Atten Disord 12: 410–421 10.1177/1087054708315065 18400983

[46] Lange KW, Tucha L, Walitza S, Gerlach M, Linder M, et al.. (2007) Interaction of attention and graphomotor functions in children with attention deficit hyperactivity disorder. J Neural Transm Suppl: 249–259. 10.1007/978-3-211-73574-9_31 17982901

[47] Dinn WM, Robbins NC, Harris CL (2001) Adult attention-deficit/hyperactivity disorder: neuropsychological correlates and clinical presentation. Brain and Cognition 46: 114–121. 11527308 10.1016/s0278-2626(01)80046-4

[48] Boonstra AM, Oosterlann J, Sergeant JA, Buitelaar JK (2005) Executive functioning in adult ADHD: a meta-analytic review. Psychol Med 35: 1097–1108 10.1017/S003329170500499X 16116936

[49] Tucha O, Mecklinger L, Laufkötter R, Kaunzinger I, Paul GM, et al (2005) Clustering and switching on verbal and figural fluency functions in adults with attention deficit hyperactivity disorder. PCNP 10: 231–248 10.1080/13546800444000047 16571461

[50] Biederman J, Fried R, Petty CR, Wozniak J, Doyle AE, et al (2010) Cognitive Development in Adults With Attention-Deficit/Hyperactivity Disorder. J Clin Psychiatry 72: 11–16 10.4088/JCP.09m05420pur 21034681

[51] Ivanchak N, Abner EL, Carr SA, Freeman SJ, Seybert A, et al (2011) Attention-deficit/hyperactivity disorder in childhood is associated with cognitive test profiles in the geriatric population but not with mild cognitive impairment or Alzheimer’s disease. J Aging Res 2011: 729801 10.4061/2011/729801 21822493 PMC3142705

[52] Das D, Cherbuin N, Anstey KJ, Easteal S (2012) ADHD Symptoms and Cognitive Abilities in the Midlife Cohort of the PATH Through Life Study. J Atten Disord. doi:10.1177/1087054712460887. 10.1177/1087054712460887 23223123

[53] Anstey KJ, Christensen H, Butterworth P, Easteal S, Mackinnon A, et al (2012) Cohort profile: the PATH through life project. Int J Epidemiol 41: 951–960 10.1093/ije/dyr025 21349904

[54] Kessler RC, Adler L, Ames M, Demler O, Faraone S, et al (2005) The World Health Organization Adult ADHD Self-Report Scale (ASRS): a short screening scale for use in the general population. Psychol Med 35: 245–256 10.1017/S0033291704002892 15841682

[55] Kessler RC, Adler LA, Gruber MJ, Sarawate CA, Spencer T, et al (2007) Validity of the World Health Organization Adult ADHD Self-Report Scale (ASRS) Screener in a representative sample of health plan members. Int J Methods Psychiatr Res 16: 52–65 10.1002/mpr.208 17623385 PMC2044504

[56] Folstein MF (1983) The Mini-Mental State Examination. Arch Gen Psychiatry 40: 812–812 10.1001/archpsyc.1983.01790060110016 6860082

[57] Hesse M (2013) The ASRS-6 Has Two Latent Factors: Attention Deficit and Hyperactivity. J Atten Disord 17: 203–207 10.1177/1087054711430330 22262467

[58] Martin A, Rief W, Klaiberg A, Braehler E (2006) Validity of the Brief Patient Health Questionnaire Mood Scale (PHQ-9) in the general population. Gen Hosp Psychiatry 28: 71–77 10.1016/j.genhosppsych.2005.07.003 16377369

[59] Spitzer RL, Kroenke K, Williams JB (1999) Validation and utility of a self-report version of PRIME-MD: the PHQ primary care study. Primary Care Evaluation of Mental Disorders. Patient Health Questionnaire. JAMA 282: 1737–1744. 10568646 10.1001/jama.282.18.1737

[60] Baddeley A, Emslie H, Nimmo-Smith I (1993) The Spot-the-Word test: a robust estimate of verbal intelligence based on lexical decision. Br J Clin Psychol 32: 55–65 10.1111/j.2044-8260.1993.tb01027.x 8467274

[61] Sánchez-Cubillo I, Periáñez JA, Adrover-Roig D, Rodríguez-Sánchez JM, Ríos-Lago M, et al (2009) Construct validity of the Trail Making Test: role of task-switching, working memory, inhibition/interference control, and visuomotor abilities. J Int Neuropsychol Soc 15: 438–450 10.1017/S1355617709090626 19402930

[62] Reitan RM, Wolfson D (1993) The Halstead-Reital Neuropsychology Test Battery: Theory and Clinical Interpretation. Second. Tucson: Neuropsychology Press.

[63] Smith A (1982) Symbol Digit Modality Test Manual. Los Angeles: Western Psychological Services.

[64] Delis DC, Kramer JH, Kaplan E, Ober BA (1987) California Verbal Learning Test: Adult Version. San Antonio: The Psychological Corporation.

[65] Wechsler D (1945) Wechsler memory scale manual. New York : The Psychological Corporation.

[66] Welford AT (1988) Reaction time, speed of performance, and age. Ann N Y Acad Sci 515: 1–17. 10.1111/j.1749-6632.1988.tb32958.x 3364878

[67] Byrne BM (2004) Testing for Multigroup Invariance Using AMOS Graphics: A Road Less Traveled. Structural Equation Modeling: A Multidisciplinary Journal 11: 272–300 10.1207/s15328007sem11028

[68] Browne WM, Cudek R (1993) Alternate ways of assessing model fit. In: Bollen KA, Long JS, editors. Testing Structural Equation Models. Newbury Park: SAGE Publications, Incorporated. 136–162.

[69] Joreskog KG, Sorbom D (1984) LISREL VI user’s guide. Scientific Software.

[70] Bentler PM (1990) Comparative fit indexes in structural models. Psychol Bull 107: 238–246 10.1037/0033-2909.107.2.238 2320703

[71] Akaike H (1973) Information Theory and an extension of the maximum likelihood principle. In: Petrov BN, Csaki F, editors. *Proceedings of the 2nd International Symposium on Information Theory*. Budapest: Akademiai Kaido. 267–281.

[72] Browne MW, Cudeck R (1989) Single Sample Cross-Validation Indices for Covariance Structures. Multivariate Behavioral Research 24: 445–455 10.1207/s15327906mbr24044 26753509

[73] Sobanski E (2006) Psychiatric comorbidity in adults with attention-deficit/hyperactivity disorder (ADHD). Eur Arch Psychiatry Clin Neurosci 256 Suppl 1i26–i31 10.1007/s00406-006-1004-4 16977548

[74] Barkley RA, Guevremont DC, Anastopoulos AD, DuPaul GJ, Shelton TL (1993) Driving-related risks and outcomes of attention deficit hyperactivity disorder in adolescents and young adults: a 3- to 5-year follow-up survey. Pediatrics 92: 212–218. 8337019

[75] Das D, Cherbuin N, Butterworth P, Anstey KJ, Easteal S (2012) A population-based study of attention deficit/hyperactivity disorder symptoms and associated impairment in middle-aged adults. PLoS ONE 7: e31500 10.1371/journal.pone.0031500 22347487 PMC3275565

[76] de Zwaan M, Gruß B, Müller A, Graap H, Martin A, et al (2012) The estimated prevalence and correlates of adult ADHD in a German community sample. Eur Arch Psychiatry Clin Neurosci 262: 79–86 10.1007/s00406-011-0211-9 21499942

[77] Simon V, Czobor P, Bálint S, Mészáros A, Bitter I (2009) Prevalence and correlates of adult attention-deficit hyperactivity disorder: meta-analysis. Br J Psychiatry 194: 204–211 10.1192/bjp.bp.107.048827 19252145

[78] Berry CA, Shaywitz SE, Shaywitz BA (1985) Girls with attention deficit disorder: a silent minority? A report on behavioral and cognitive characteristics. Pediatrics 76: 801–809. 4058990

[79] Gershon J (2002) A meta-analytic review of gender differences in ADHD. J Atten Disord 5: 143–154 10.1177/108705470200500302 11911007

[80] Murphy K, Barkley R (1996) Prevalence of DSM-IV symptoms of ADHD in adult licensed drivers: Implications for clinical diagnosis. J Atten Disord 1: 147–161 10.1177/108705479600100303

[81] Aycicegi-Dinn A, Dervent-Ozbek S, Yazgan Y, Bicer D, Dinn WM (2011) Neurocognitive correlates of adult attention-deficit/hyperactivity disorder in a Turkish sample. Atten Defic Hyperact Disord 3: 41–52 10.1007/s12402-010-0050-y 21432617

[82] Moss F, Ward LM, Sannita WG (2004) Stochastic resonance and sensory information processing: a tutorial and review of application. Clin Neurophysiol 115: 267–281. 14744566 10.1016/j.clinph.2003.09.014

[83] Willcutt EG, Doyle AE, Nigg JT, Faraone SV, Pennington BF (2005) Validity of the executive function theory of attention-deficit/hyperactivity disorder: a meta-analytic review. Biol Psychiatry 57: 1336–1346 10.1016/j.biopsych.2005.02.006 15950006

[84] Brewin CR, Andrews B, Gotlib IH (1993) Psychopathology and early experience: A reappraisal of retrospective reports. Psychol Bull 113: 82–98 10.1037/0033-2909.113.1.82 8426875

[85] Neugebauer R, Ng S (1990) Differential recall as a source of bias in epidemiologic research. J Clin Epidemiol 43: 1337–1341 10.1016/0895-4356(90)90100-4 2254770

[86] Manea L, Gilbody S, McMillan D (2012) Optimal cut-off score for diagnosing depression with the Patient Health Questionnaire (PHQ-9): a meta-analysis. CMAJ 184: E191–E196 10.1503/cmaj.110829 22184363 PMC3281183

[87] Ramos-Quiroga JA, Daigre C, Valero S, Bosch R, Gómez-Barros N, et al (2009) Validation of the Spanish version of the attention deficit hyperactivity disorder adult screening scale (ASRS v. 1.1): a novel scoring strategy. Rev Neurol 48: 449–452. 19396760

[88] Zohar AH, Konfortes H (2010) Diagnosing ADHD in Israeli adults: the psychometric properties of the adult ADHD Self Report Scale (ASRS) in Hebrew. Isr J Psychiatry Relat Sci 47: 308–315. 21270505

