# Supplementary figures and images for: Attention Deficit/Hyperactivity Disorder Symptoms and Cognitive Abilities in the Late-Life Cohort of the PATH through Life Study
Source: PLoS One. 2014 Jan 28;9(1):e86552. doi: 10.1371/journal.pone.0086552 (PMC3904910; doi:10.1371/journal.pone.0086552)

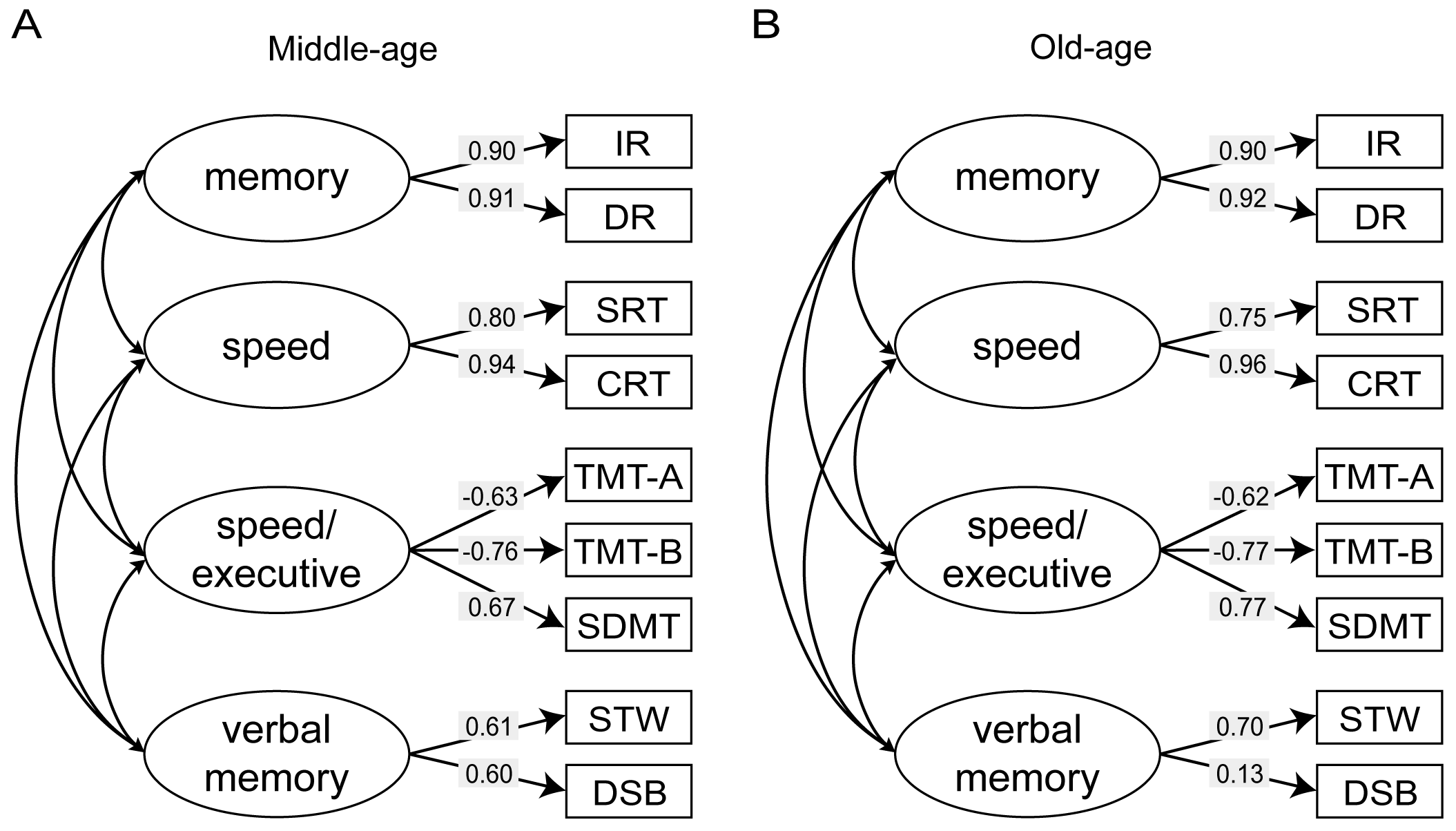

Supplement: Figure S1 — Models representing the latent structure of the cognitive tests used in the study in the MA (A) and OA (B) cohorts. Standardised factor loadings are shown. IR: Immediate Recall; DR: Delayed Recall; SRT: Simple Reaction Time; CRT: Choice Reaction Time; TMT-A: Trail Making Test A; TMT-B: Trail Making Test B; SDMT: Symbol-Digit Modalities Test; STW: Spot The Word test; DSB: Digit Span Backwards. (TIF) [file pone.0086552.s001.tif]

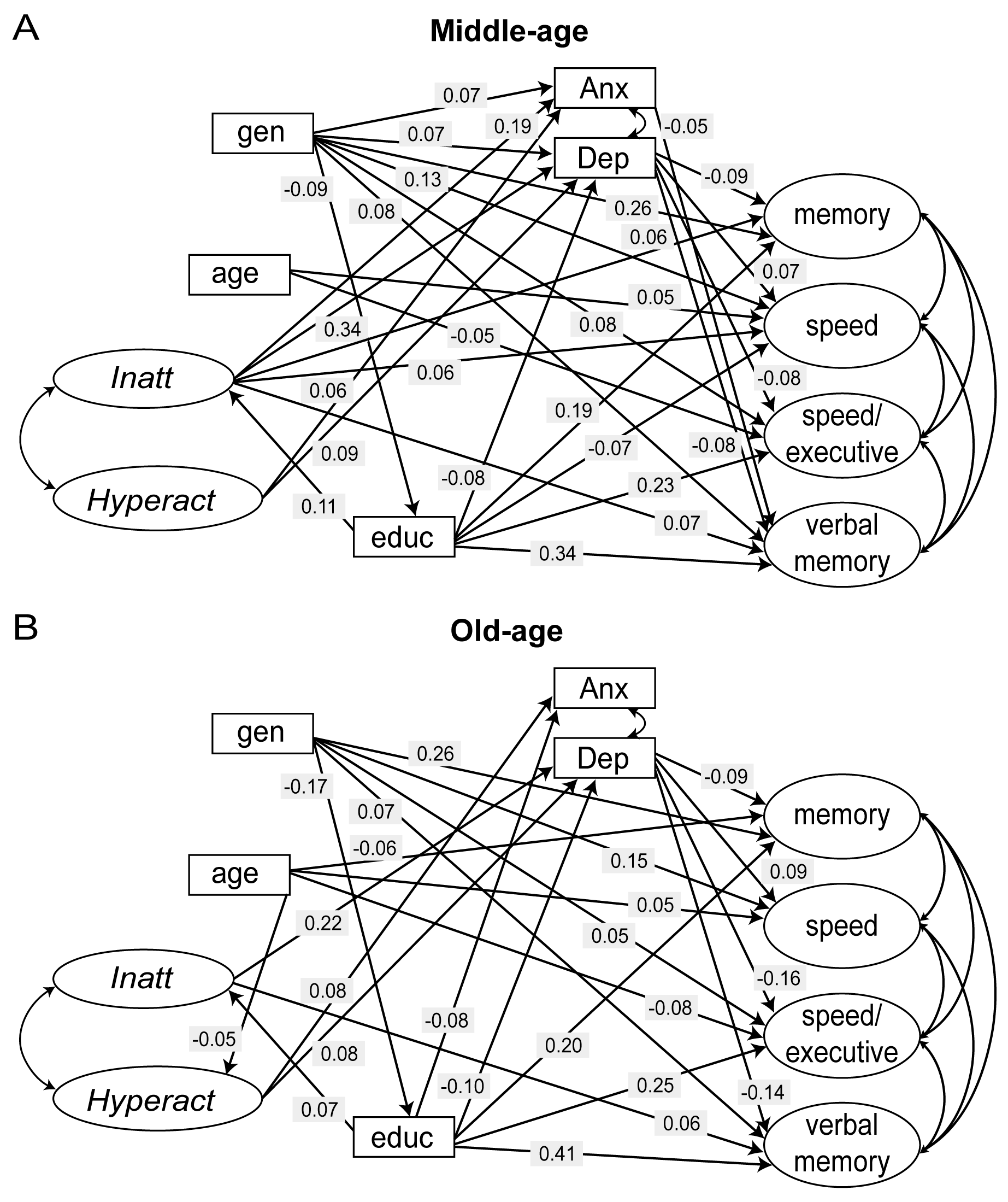

Supplement: Figure S2 — Final model for ADHD symptom–cognition analyses for MA (A) and OA (B) cohorts. Only paths significant at p<0.05 are shown. Arrows reflect direction of relationships between variables. Standardised regression coefficients are shown. gen: gender; educ: education; Inatt: latent factor Inattention; Hyperact: latent factor Hyperactivity; DEP: depression symptom measure; ANX: anxiety symptom measure. Indicators of the latent variables are not shown for the sake of clarity. (TIF) [file pone.0086552.s002.tif]
