# Supplementary material for: Attention Deficit/Hyperactivity Disorder Symptoms and Cognitive Abilities in the Late-Life Cohort of the PATH through Life Study
Source: PLoS One. 2014 Jan 28;9(1):e86552. doi: 10.1371/journal.pone.0086552 (PMC3904910; doi:10.1371/journal.pone.0086552)
Supplement: File S1 — Tables S1–S5. (DOCX) [file pone.0086552.s003.docx]

Table S1: Items in the ASRS questionnaire

| ASRS items | |
| --- | --- |
| *Inattention* | |
|  | How often do you have trouble wrapping up the fine details of a project, once the challenging parts have been done? |
|  | How often do you have difficulty getting things in order when you have to do a task that requires organization? |
|  | When you have a task that requires a lot of thought, how often do you avoid or delay getting started? |
|  | How often do you have problems remembering appointments or obligations? |
| *Hyperactivity* | |
|  | How often do you fidget or squirm with your hands or your feet when you have to sit down for a long time? |
|  | How often do you feel overly active and compelled to do things, like you were driven by a motor? |
| ASRS: adult ADHD Self-Report Scale | |

Table S2: Description of cognitive tests used

| Cognitive Test | Brief description | Construct | Reference |
| --- | --- | --- | --- |
| Spot-the-Word Test | Participants were presented with a pair of items comprising of one word and one non-word and were required to identify the word. | verbal ability | [1] |
| Trail Making Test | Participant were required to ‘connect the dots’ of 25 consecutive targets. There are two parts to the test. In Part A the targets are numerical and have to be connected in an ascending order (as 1-2-3-4-...). In Part B, the target alternates between numbers and letters (1-A-2-B-3-C-…) | Part A: visual attention  Part B: task-switching | [2,3] |
| Simple and Choice Reaction Time | Simple and Choice Reaction Time tasks were administered using a hand held box with two depressible buttons (left and right) and two red stimulus lights and one get-ready light. For the Simple reaction Time, one of the stimulus lights was activated and participants were instructed to press the right hand button (regardless of dominance). For the Choice Reaction Time, participants had to press the button corresponding to the left or the right stimulus light. Simple reaction Time was measured first using four blocks of 20 trials followed by two blocks of 20 trials for Choice Reaction Time. | information processing speed | [4] |
| California Verbal Learning Test (first trial) | Participants were presented with a ‘shopping list’ of 16 common words from four different categories and were asked to recall as many of these items as possible either immediately after the list has been presented or after a short delay. | verbal memory | [5] |
| Symbol-Digit Modalities Test | Participants were required to connect basic roman numerals with a series of geometric shapes using a reference key. | information processing speed | [6] |
| Digits Span Backwards | Participants were presented with a series of digits (‘3, 6, 8’) and were required to repeat them in reverse order. If participants responded correctly then a longer sequence of digits was presented. | working memory | [7] |

Table S3: Pearson correlations coefficients between ASRS latent factors and cognitive test measures for MA cohort.

|  | *Inatt* | | *Hyperact* | STW | TMT-A | TMT-B | SRT | CRT | IR | DR | SDMT | DSB |
| --- | --- | --- | --- | --- | --- | --- | --- | --- | --- | --- | --- | --- |
| *Inatt* | - | | .320^**^ | .134^**^ | .063^**^ | .013 | .029 | .085^**^ | .031 | .026 | -.035 | .037 |
| *Hyperact* |  | | - | .012 | -.010 | .005 | -.005 | .009 | .027 | .049^*^ | -.001 | .016 |
| STW |  | |  | - | -.220^**^ | -.346^**^ | -.135^**^ | -.140^**^ | .260^**^ | .264^**^ | .288^**^ | .365^**^ |
| TMT-A |  | |  |  | - | .493^**^ | .248^**^ | .288^**^ | -.177^**^ | -.184^**^ | -.431^**^ | -.209^**^ |
| TMT-B |  | |  |  |  | - | .219^**^ | .259^**^ | -.238^**^ | -.248^**^ | -.500^**^ | -.364^**^ |
| SRT |  | |  |  |  |  | - | .752^**^ | -.137^**^ | -.114^**^ | -.200^**^ | -.117^**^ |
| CRT |  | |  |  |  |  |  | - | -.131^**^ | -.107^**^ | -.320^**^ | -.136^**^ |
| IR |  | |  |  |  |  |  |  | - | .814^**^ | .281^**^ | .225^**^ |
| DR |  | |  |  |  |  |  |  |  | - | .303^**^ | .200^**^ |
| SDMT |  | |  |  |  |  |  |  |  |  | - | .299^**^ |
| DSB |  | |  |  |  |  |  |  |  |  |  | - |
| * *p*<0.05  ** *p*<0.01  *Inatt* and *Hyperact*: ASRS latent factors; STW: Spot The Word test; TMT-A: Trail Making Test A; TMT-B: Trail Making Test B; SRT: Simple Reaction Time; CRT: Choice Reaction Time; IR: Immediate Recall; DR: Delayed Recall; SDMT: Symbol-Digit Modalities Test; DSB: Digit Span Backwards | | | | | | | | | | | | |
|  | |  | | | | | | | | | | |

Table S4: Pearson correlations coefficients ASRS latent factors and cognitive test measures for OA cohort.

|  | *Inatt* | | *Hyperact* | STW | TMT-A | TMT-B | SRT | CRT | IR | DR | SDMT | DSB |
| --- | --- | --- | --- | --- | --- | --- | --- | --- | --- | --- | --- | --- |
| *Inatt* | - | | .350^**^ | .092^**^ | .027 | .005 | .007 | .042 | -.022 | -.036 | -.053^*^ | .017 |
| *Hyperact* |  | | - | .038 | -.019 | -.036 | .025 | .019 | -.016 | -.026 | -.006 | -.007 |
| STW |  | |  | - | -.150^**^ | -.321^**^ | -.176^**^ | -.150^**^ | .264^**^ | .261^**^ | .321^**^ | .089^**^ |
| TMT-A |  | |  |  | - | .497^**^ | .187^**^ | .245^**^ | -.183^**^ | -.188^**^ | -.473^**^ | -.053^*^ |
| TMT-B |  | |  |  |  | - | .236^**^ | .293^**^ | -.246^**^ | -.259^**^ | -.591^**^ | -.059^*^ |
| SRT |  | |  |  |  |  | - | .724^**^ | -.119^**^ | -.121^**^ | -.223^**^ | -.030 |
| CRT |  | |  |  |  |  |  | - | -.098^**^ | -.117^**^ | -.314^**^ | -.049 |
| IR |  | |  |  |  |  |  |  | - | .837^**^ | .299^**^ | .032 |
| DR |  | |  |  |  |  |  |  |  | - | .311^**^ | .037 |
| SDMT |  | |  |  |  |  |  |  |  |  | - | .066^*^ |
| DSB |  | |  |  |  |  |  |  |  |  |  | - |
| * *p*<0.05  ** *p*<0.01  *Inatt* and *Hyperact*: ASRS latent factors; STW: Spot The Word test; TMT-A: Trail Making Test A; TMT-B: Trail Making Test B; SRT: Simple Reaction Time; CRT: Choice Reaction Time; IR: Immediate Recall; DR: Delayed Recall ; SDMT: Symbol-Digit Modalities Test; DSB: Digit Span Backwards | | | | | | | | | | | | |
|  | |  | | | | | | | | | | |

Table S5: Path coefficients from multi-group SEM analyses of ADHD symptom–cognition relationships.

|  | |  |  | MA | | | OA | | |
| --- | --- | --- | --- | --- | --- | --- | --- | --- | --- |
|  | |  |  | Coefficients^§^ | SE^†^ | *p* | Coefficients^§^ | SE^†^ | *p* |
| age | 🡪 | | *Hyperact* | - | - | - | -0.031 | 0.015 | 0.044 |
| gender | 🡪 | | education | -0.379 | 0.101 | <0.001 | -0.866 | 0.132 | 0.003 |
| education | 🡪 | | *Inatt* | 0.028 | 0.005 | <0.001 | 0.013 | 0.005 | 0.003 |
| gender | 🡪 | | anxiety | 0.031 | 0.010 | 0.002 | - | - | - |
| education | 🡪 | | anxiety | - | - | - | -0.005 | 0.002 | 0.001 |
| *Inatt* | 🡪 | | anxiety | 0.076 | 0.013 | <0.001 | - | - | - |
| *Hyperact* | 🡪 | | anxiety | 0.014 | 0.006 | 0.006 | 0.012 | 0.006 | 0.004 |
| gender | 🡪 | | depression | 0.541 | 0.168 | 0.002 | - | - | - |
| education | 🡪 | | depression | -0.153 | 0.034 | <0.001 | -0.106 | 0.028 | <0.001 |
| *Inatt* | 🡪 | | depression | 2.419 | 0.197 | <0.001 | 1.276 | 0.165 | <0.001 |
| *Hyperact* | 🡪 | | depression | 0.350 | 0.093 | <0.001 | 0.224 | 0.092 | 0.004 |
| age | 🡪 | | memory | - | - | - | -0.033 | 0.012 | 0.005 |
| gender | 🡪 | | memory | 0.425 | 0.037 | <0.001 | 0.443 | 0.041 | <0.001 |
| education | 🡪 | | memory | 0.071 | 0.008 | <0.001 | 0.063 | 0.008 | <0.001 |
| *Inatt* | 🡪 | | memory | 0.081 | 0.032 | 0.012 | - | - | - |
| depression | 🡪 | | memory | -0.019 | 0.006 | <0.001 | -0.027 | 0.007 | <0.001 |
| age | 🡪 | | speed | 0.018 | 0.008 | 0.017 | 0.020 | 0.010 | 0.039 |
| gender | 🡪 | | speed | 0.138 | 0.023 | <0.001 | 0.184 | 0.031 | <0.001 |
| education | 🡪 | | speed | -0.016 | 0.006 | 0.003 | - | - | - |
| *Inatt* | 🡪 | | speed | 0.055 | 0.021 | 0.005 | - | - | - |
| depression | 🡪 | | speed | 0.009 | 0.003 | 0.003 | 0.020 | 0.006 | <0.001 |
| age | 🡪 | | speed/exec | -0.018 | 0.004 | <0.001 | -0.032 | 0.007 | <0.001 |
| gender | 🡪 | | speed/exec | 0.089 | 0.025 | <0.001 | 0.061 | 0.031 | 0.050 |
| education | 🡪 | | speed/exec | 0.061 | 0.006 | <0.001 | 0.060 | 0.006 | <0.001 |
| depression | 🡪 | | speed/exec | -0.011 | 0.004 | 0.002 | -0.036 | 0.008 | <0.001 |
| gender | 🡪 | | verbal memory | 0.067 | 0.021 | <0.001 | 0.006 | 0.002 | 0.004 |
| education | 🡪 | | verbal memory | 0.072 | 0.005 | <0.001 | 0.007 | <0.001 | <0.001 |
| *Inatt* | 🡪 | | verbal memory | 0.057 | 0.009 | <0.001 | 0.006 | 0.001 | <0.001 |
| anxiety | 🡪 | | verbal memory | -0.109 | 0.051 | 0.030 | - | - | - |
| depression | 🡪 | | verbal memory | -0.010 | 0.005 | 0.001 | -0.002 | <0.001 | <0.001 |
| Only paths significant at *p*<0.05 are shown  § unstandardised estimate  † standard errors were computed from 2000 bootstrap-resampled datasets  MA: middle-age cohort; OA: older-age cohort; *Inatt*: latent factor Inattention; *Hyperact*: latent factor Hyperactivity;  Cognition latent factors include memory, speed, speed/executive function, verbal memory  χ^2^ =43.789, *df*=40, *p*=0.314; RMSEA= 0.005; AGFI=0.992; CFI=1.0; AIC=227.789; BCC=229.098 | | | | | | | | | |

**References**

1. Baddeley A, Emslie H, Nimmo-Smith I (1993) The Spot-the-Word test: a robust estimate of verbal intelligence based on lexical decision. Br J Clin Psychol 32: 55–65. doi:10.1111/j.2044-8260.1993.tb01027.x.

2. Reitan RM, Wolfson D (1993) The Halstead-Reital Neuropsychology Test Battery: Theory and Clinical Interpretation. Second. Tucson: Neuropsychology Press.

3. Sánchez-Cubillo I, Periáñez JA, Adrover-Roig D, Rodríguez-Sánchez JM, Ríos-Lago M, et al. (2009) Construct validity of the Trail Making Test: role of task-switching, working memory, inhibition/interference control, and visuomotor abilities. J Int Neuropsychol Soc 15: 438–450. doi:10.1017/S1355617709090626.

4. Welford AT (1988) Reaction time, speed of performance, and age. Ann N Y Acad Sci. 515: 1-17.

5. Delis DC, Kramer JH, Kaplan E, Ober BA (1987) California Verbal Learning Test: Adult Version. San Antonio: The Psychological Corporation.

6. Smith A (1982) Symbol Digit Modality Test Manual. Los Angeles: Western Psychological Services.

7. Wechsler D (1945) Wechsler memory scale manual. New York: The Psychological Corporation.
